# The IPA, a Modified Numerical System for Pain Assessment and Intervention

**DOI:** 10.5435/JAAOSGlobal-D-21-00174

**Published:** 2021-09-02

**Authors:** Rahul Vaidya, Austen Washington, Sasha Stine, Andreea Geamanu, Ian Hudson

**Affiliations:** From the Orthopaedic Surgery Department, Wayne State University, Detroit Medical Center, Detroit, MI (Dr. Vaidya); Orthopaedic Surgery Department, Detroit Medical Center Cardiovascular Institute, Detroit, MI (Mr. Washington and Dr. Stine); Orthopaedic Surgery Department, Detroit Medical Center, Detroit, MI (Dr. Geamanu); and US Army Institute of Surgical Research, Fort Sam Houston, TX (Dr. Hudson).

## Abstract

The objective of this study was to (1) construct a pain scale that improves communication between healthcare providers and patients (Interventional Pain Assessment [IPA] tool) and (2) to validate this new pain scale with the numeric rating scale of 0 to 10 Numerical Rating System (NRS).

**Methods:**

The IPA uses only three categories: 0 = “I have no pain,” 1 = “My pain is tolerable (no intervention needed),” and 2 = “my pain is intolerable, (intervention needed).” An Institutional Review Board–approved study was done on 322 consecutive patients who were recovering from fracture treatment. We compared ratings of the IPA with NRS. We also asked patients which scale they preferred. Statistical analysis included Kendall rank correlation (Kendall τ) and Spearman rho to determine correlation with the NRS.

**Results:**

The IPA exhibited a statistically significant association with the NRS (τ = 0.58, *P* < 0.0001). Discordant answers were provided by 23.6% patients; 4.7% regarded their mild-to-moderate pain as intolerable (15/322) while 18.9% reported their severe pain as tolerable (61/322). Eighty-two percent of patients preferred the IPA.

**Conclusion:**

The IPA is a valid pain scale and has exhibited strong correlation with the NRS 0 to 10, displays simple minimally clinical important difference calculation, and provides meaningful information on the effect of pain control modulation.

Pain is a unique subjective experience, and the medical treatment of pain has been an issue in the spotlight of medical research in recent decades.^1-3^ Pain scales were introduced to allow physicians to quantify this subjective experience in a way that would allow them to effectively manage pain. The study of pain treatment and the use of pain scales date back to the early 1930s, with minor advancements in assessment tools and treatment practices over the century.^4^ In 2001, the Joint Commission introduced new standards and guidelines for pain assessment and management in an effort to address the inadequate treatment of pain in hospitals nationwide.^5^ As a result, pain has been heightened to the point of being assessed along with the other vital signs commonly taken on a visit to a physician. Medical practitioners rely on pain scales as a means of accurately surveilling pain to assist in the management and treatment of pain.^6^

The most commonly used scales are the Visual Analog Scale, Verbal Rating System, Numerical Rating System (NRS), and the Faces Pain Scale–Revised. These scales have been validated for use with a diverse patient population for the unidimensional assessment of the severity of pain in a variety of clinical settings.^7,8,9,10,11^ Unidimensional scales such as the NRS are the preferred type of pain scale because of their time efficiency, simplicity, patient preference, ability to determine the effectiveness of analgesic treatments, and sensitivity to incremental changes in the intensity of pain.^4,12,13,14,15,16,17^

Despite the widespread use of unidimensional scales in clinical settings, discrepancies still exist between the self-assessed pain scores of patients and their medical professionals’ assessment.^16,18,19,20,21^ More specifically, studies have shown that medical professionals have a tendency to underscore patients’ pain.^17,22,23^

The implementation of pain scales has led to moderate improvements in pain assessment and management. However, several studies demonstrate that patients remain dissatisfied with their pain treatment.^24-26^ In a recent study on patients' perception of the NRS, patients expressed that it was not only difficult to provide a numerical rating of their pain but also that a number of patients failed to accurately describe their pain experience.^27^ The movement toward more precise scales to incorporate every possible response is not a feasible option because of limited medical field resources, administrative burden, and financial limitation associated with such an endeavor and the infinite locations, severity, and descriptors of pain. Therefore, in this study, we aim to validate a new pain scale through correlation analysis with the already validated NRS. This pain scale uses only three options: no pain (0), tolerable pain (1), and intolerable pain (2). This scale has no ambiguity and has a direct relationship with treatment. Patients who have no pain or tolerable pain have adequate pain control, and those who have intolerable pain require intervention. We hypothesize that the no pain-tolerable pain-intolerable pain scale will show moderate-to-strong correlations with the NRS.

## Methods

### Participants

This study was approved by the Institutional Review Board. Data were obtained through an interview style of questioning from 354 patients at an orthopaedic fracture clinic at a level-one ACS-verified US trauma center. Participants consisted of follow-up fracture patients attending clinic in the orthopaedic department because they were likely to still have pain. Exclusion criteria included those who were cognitively impaired, those who spoke a language other than English without an interpreter present, or refusal to participate in the study. Informed consent was obtained before the interview.

### Numeric Rating Scale

The NRS is a validated 11-point unidimensional numerical scale that measures the intensity of pain.^28^ The scale can be administered verbally or graphically to be completed by the patient; in both cases, patients are required to rate their pain from 0 to 10, with 0 being no pain and 10 being the worst pain imaginable.^11^ We designated the categorical cutoff points for the NRS as mild (1–4), moderate (5–6), and severe (7 to 10), as previously reported.^17,29,30^

### Intervention Pain Assessment Scale

The proposed scale is a 3-point unidimensional scale that is used to assess the general intensity of pain. The scale is administered verbally or as a paper graphic. The responses are no pain, tolerable pain, and intolerable pain. No pain corresponds with a score of 0, tolerable pain is represented with a score of 1, and intolerable pain is given a score of 2.

### Procedure

Age and sex of the patient, International Statistical Classification of Diseases and Related Health Problems (ICD) 10 code, and time from fracture surgery were recorded. Patients were asked to report their level of pain on a standard 0 to 10 NRS and the Interventional Pain Assessment (IPA): 0 as no pain, 1 as tolerable pain, or 2 as intolerable pain. This process was alternated between successive patients. Patients were also asked which scale they preferred. Discordance between IPA and NRS was grossly defined as those who described mild or moderate pain (NRS 1 to 6) as intolerable or severe pain (7 to 10) as tolerable. Given the nonnormal arrangement of NRS, a low number of possible ordinal values in the IPA scale and the inevitable frequency of ties, Kendall rank correlation (Kendall τ) and Spearman rho were selected to serve as measures of association. A *P* value of <0.05 was considered to be significant. Statistical analysis was done using SAS 9.4 (SAS Institute).

## Results

Of the 354 patients, 32 patients surveyed were unable to provide a response for either one or both of the pain scales and two patients were excluded because of language barrier that made informed consent impossible to obtain.

That left 320 patients who were interviewed for this study. The age of participants ranged from 18 to 97 years with a mean of 50.25 + 15.48. Among the patients interviewed, 151 (47%) were female and 171 (53%) were male.

**Figure 1:**
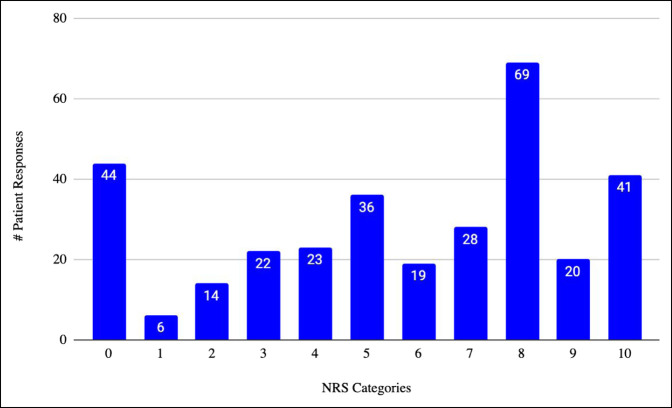
Numeric rating scale (NRS) responses. Graph demonstrating 322 patient responses to the NRS grouped under the categories of the NRS ranging from 0 to 10. The number of responses to each category is shown at the top of each bar.

The categories of responses to the NRS are shown in Figure 1. The mean pain score on the NRS was 5.66, with a SD of 3.24. Forty-four participants (14%) rated themselves as having no pain, 65 participants (20%) rated their pain as mild (mean 2.95 + 0.97), 55 participants (17%) rated their pain as moderate (mean 5.35 + 0.48), and 158 participants (49%) rated their pain as severe (mean 8.47 + 1.06).

**Figure 2:**
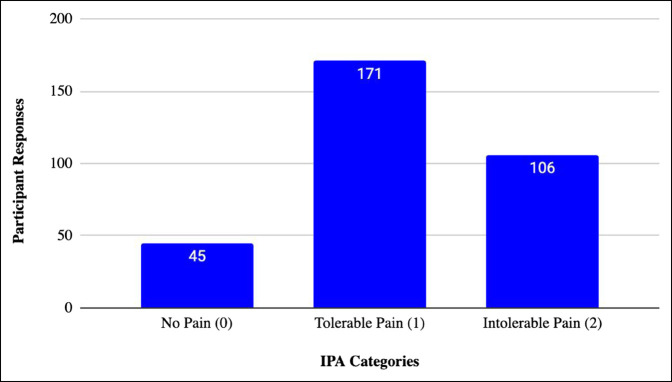
Intervention pain assessment (IPA) responses. Graph demonstrating 322 patient responses to the IPA grouped under the following categories for the IPA: no pain (0), tolerable pain (1), and intolerable pain (2). The number of responses to each category is shown at the top of each bar.

The mean score of the tolerable-intolerable scale was 1.19 + 0.66. Responses to the IPA can be seen in Figure 2. There were 45 responses with no pain (0), 171 with tolerable pain (1), and 106 with intolerable pain (2). Mean responses to both scales including SD are provided in Table 1.

**Table 1:** Means, Medians, Ranges, and SDs of the NRS and the IPA

	Mean	Median	Range	SD
NRS	5.66	6	0-10	3.24
Mild	2.95	3	1-3	0.97
Moderate	5.35	5	4-6	0.48
Severe	8.47	8	7-10	1.06
IPA	1.16	1	0-2	0.66

IPA = Intervention Pain Assessment, NRS = Numeric Rating Scale

The hypothesis of this study was that pain recorded on the NRS as mild and moderate would correlate with tolerable pain and pain recorded as severe would correlate with intolerable pain. Based on the results recorded from the patients in this study, 76 patients (23.6%) reported discordant pain scores. In total, 15 patients (4.7%) who rated their pain as mild or moderate (≤6) also rated their pain as intolerable while 61 patients (18.9%) who rated their pain as severe (≥7) described their pain as tolerable (Supplemental Content 1, http://links.lww.com/JG9/A157). In 246 cases (76.4%), patients reported their pain in concordance with both scales, and although still a majority, this suggests that the NRS may not be suitable in accurately describing pain intensity.

**Figure 3:**
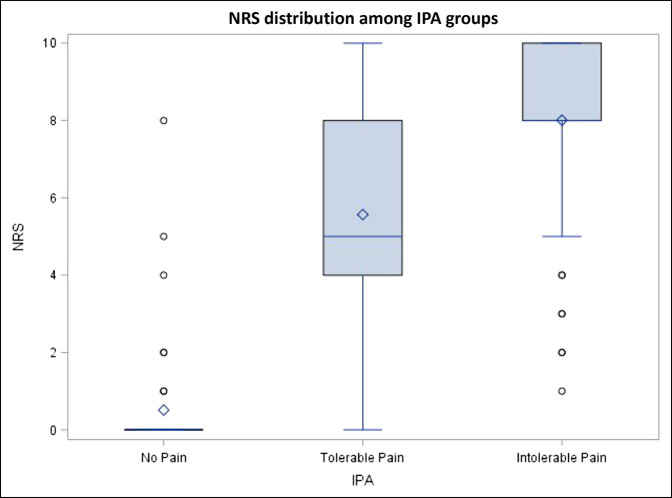
Box plot demonstrating the interquartile ranges of the responses of the NRS distribution among IPA groups (no pain, tolerable pain, and intolerable pain). Points exhibited outside of the interquartile ranges for no pain and intolerable pain represent response outliers. Although difficult to see the median response for, the no pain is included in the box plot and is 0. IPA = Intervention Pain Assessment, NRS = Numeric Rating Scale

A statistically significant association was found between NRS and IPA (τ = 0.58, *P* < 0.0001), and clusters of NRS were generally concordant with IPA (Figure 3). This study included the follow-up occurred at a median of 12 weeks (IQR 8 to 28 weeks). No association was found between time and either NRS (0.034, *P* < 0.52) or IPA (0.01, *P* < 0.84) (Supplemental Content 2, http://links.lww.com/JG9/A158). Seventy-five percent of patients who rated their pain as mild according to the NRS (1 to 4) rated their pain as tolerable according to the IPA. Seventy-six percent of patients who rated their pain as moderate (5 to 6) also rated their pain as tolerable. Sixty-four percent of patients who rated their pain as severe (8 to 10) also rated their pain as intolerable. There is a pattern of proportionately more patients who rate their pain as mild or moderate also rating their pain as tolerable; however, a deviation occurs when patients begin ranking their pain as a 7 on the NRS and intolerable on the IPA. This deviation continues to occur because proportionately fewer patients who rate their pain as severe according to the NRS rate their pain as intolerable in comparison to the average percentage of patients rating their pain as mild or moderate and tolerable. There were relatively similar percentages of patients in the mild, moderate, and severe category who also rate their pain as tolerable. This closeness in values would indicate that the NRS is a poor descriptor of actual pain tolerance and patient pain experience.

Eighty-two percent of patients preferred the IPA scale because it was understandable and thought they conveyed their pain situation. Twelve percent were neutral, and six percent preferred the NRS.

## Discussion

The NRS is a validated tool for assessing the severity of pain the patient is experiencing; however, the hypothesis of this study questions its utility and accuracy in providing a clear depiction of that severity because of the arbitrary numerical system. The assumptions that arise from a high or low score on the NRS have the potential to lead to erroneous pain prescriptions. Because of the highly subjective nature of pain and the pain experience, a fundamental problem arises when the patient is confined to a nondescriptive unidimensional scale and the physician heavily relies on the response to this scale to guide the pain management process. Studies have highlighted how the responses to the NRS are not enough to aid in pain treatment.^24,31-33^ A recent study describing patients' feelings toward the NRS exhibits the discrepancies in scoring because of the fact that patients incorporate more factors outside of pain when selecting a score. Patients expressed a fear of not being believed because of a higher pain rating. This study also found that a wide range of pain scores denoted when the pain was unbearable.^27^ A patient who rates their pain as 5 of 10, for example, may deem that pain as bearable; however, that same pain intensity could possibly be considered as unbearable pain for another patient. They also communicated a lack of understanding of how physicians used this arbitrary number in pain management and feared that it was compared with other patients’ pain scores.^27^ Some patients fear selecting a number on the various unidimensional scales that does not provide them with adequate pain relief and will force them to fabricate a number that would allow them to receive more medication.^18^ This thought process may put patients at risk for poor pain management due to the potential for biases that arise because of a physician’s skepticism in the interpretation of an NRS score.^40^ Ultimately, the unidimensional nature of the scale becomes compromised when patients’ pain scores are influenced by aspects other than pain. This can place a burden on the physician when interpreting the score in determining the effectiveness of pain medication and not knowing whether the medication is failing to treat pain or symptoms related to but not directly caused by pain.

Several attempts have been made to introduce different methods of evaluating pain and directing its treatment. Vangronsveld and Linton revealed that simply using language that validates rather than judges or dismisses the patient’s pain experience can lead to notable improvement in psychological and physiological symptoms that exist with chronic pain.^33^ Topham and Drew invented a new pain assessment tool, named the Clinically Aligned Pain Assessment, to completely replace the NRS. They also took into account the ability to tolerate pain, and they associated this with the comfort category.^32^ From a patient and family perspective, the study results describe inadequacy of a numerical scale in representation of pain intensity, sense of suffering, or the effect of the pain on patients’ functioning at home or sleep.

There are multidimensional pain scale options available that allow for a more comprehensive pain profile of the patient. Clinicians commonly use the McGill Pain Questionnaire, Short-Form McGill Pain Questionnaire, Brief Pain Inventory, and the Global Pain Scale for a multidimensional analysis of the patient's pain, and each of the scale has been validated and used in both chronic and acute pain sufferers.^7,28,34,35,36,37,38,39^ Although multidimensional pain scales such as the McGill Pain Questionnaire offer a more substantial analysis of the physiological, psychological, and social aspects of pain to improve their content validity, studies have shown that clinicians defer to unidimensional pain scales because of the time required in administering more comprehensive scales.^12-14,16^

The IPA remedies many of the complications that arise from legacy unidimensional pain scales by providing usable information for the physician and caregivers on any needed interventions regarding the pain being experienced by the patient. The IPA scale has a small number of responses and is well-defined to the point where it minimizes the burden on the healthcare professional to interpret what the patient’s score means. The IPA measures pain as an actionable data point by giving patients an opportunity to express their tolerance to pain. It provides an actionable answer of whether pain is present and if it needs to be treated. Patients were asked about the presence of pain and their ability to tolerate it, which eliminates the need to describe their experience with an arbitrary number. Face validity of the IPA is demonstrated by the fact that the target population can recognize the type of information that is requested from them. The IPA exhibits criterion validity in the assessment of pain intensity demonstrated through Kendall rank and Spearman rho correlations. A minimally clinical important difference in score is also easy to track because the responses on the scale are limited. There is also meaning behind the values on the IPA scale as compared with the NRS. When patients say they have tolerable pain, this conveys the presence of discomfort, but not the necessity for a change to treatment. With intolerable pain, the patient is communicating that they are unhappy with their pain control and that an immediate intervention is required. In comparison, an arbitrary NRS score of 7 simply informs the physician that the patient is experiencing some level of pain; it fails to provide the physician with any real information regarding the need for an intervention, urgency, or the necessary strength of the medication. In this study, the IPA demonstrated a statistically significant correlation with the commonly used NRS, but with an important distinction, in that a nontrivial proportion of patients (nearly 1 in 5) whose pain may have been regarded as intolerable by common bias instead described their pain as tolerable. The NRS and IPA displayed agreement among 77% of patients interviewed. Although still a majority, this suggests that the NRS may not be suitable in accurately describing pain intensity.

Limitations to this study include a specific sample population; the participants were limited to an orthopaedic clinic. To generalize the findings of this study for the application of the new assessment method, the pain scale should be further evaluated with a more diverse population of participants from a wider variety of medical departments where pain is evaluated. Although unintentional, children were not present in this study and should be included in future studies for further pain scale evaluation. Additional research into the IPA scale should assess patient comprehension of response choices, as well as a longitudinal study to determine the accuracy and capability of the IPA in determining the effectiveness of medical and pharmaceutical treatments for pain, and qualitative study that reflects the opinions of clinicians on its implementation. Studies measuring cost-effectiveness and administrative burden should also be conducted to determine the viability of the IPA for nationwide implementation. Implementation of the IPA has a strong potential to increase patient satisfaction and provide a clearer understanding of the patient's experience. None of the pain scales was statistically associated with time, suggesting that the IPA operated consistently at the first follow-up and more remotely.

In conclusion, the NRS is a functional tool for assessing pain; however, the arbitrary numbers fail to capture the reality the patient is experiencing. Reliance on pain rating systems such as the NRS is an insufficient means of combating the debilitating symptoms that come with pain. Our hypothesis was validated because the IPA scale exhibited strong statistical correlations with the NRS. Its simplicity provides easy conveyance for the physician, and minimally clinical important difference is easily calculable. The IPA exhibits construct, face, and criterion validity in the assessment of pain intensity.

## Supplementary Material

SUPPLEMENTARY MATERIAL
