# Supplementary material for: The IPA, a Modified Numerical System for Pain Assessment and Intervention
Source: J Am Acad Orthop Surg Glob Res Rev. 2021 Sep 2;5(9):e21.00174. doi: 10.5435/JAAOSGlobal-D-21-00174 (PMC8415923; doi:10.5435/JAAOSGlobal-D-21-00174)
Supplement: SUPPLEMENTARY MATERIAL [file jagrr-5-e21.00174-s001.docx]

| **Supp Content 1: NRS subcategory responses placed into IPA categories** | | | |
| --- | --- | --- | --- |
| **IPA** | NRS Subcategories | # | Categorical Pop |
| No Pain (45) |  |  |  |
|  | No Pain | 38 | 84.44% |
|  | Mild | 5 | 11.11% |
|  | Moderate | 1 | 2.22% |
|  | Severe | 1 | 2.22% |
| Tolerable (171) |  |  |  |
|  | No Pain | 6 | 3.51% |
|  | Mild | 53 | 30.99% |
|  | Moderate | 46 | 26.90% |
|  | Severe | 66 | 38.60% |
| Intolerable (106) |  |  |  |
|  | No Pain | 0 | 0.00% |
|  | Mild | 7 | 6.60% |
|  | Moderate | 8 | 7.55% |
|  | Severe | 91 | 85.85% |
