# Supplementary material for: The IPA, a Modified Numerical System for Pain Assessment and Intervention
Source: J Am Acad Orthop Surg Glob Res Rev. 2021 Sep 2;5(9):e21.00174. doi: 10.5435/JAAOSGlobal-D-21-00174 (PMC8415923; doi:10.5435/JAAOSGlobal-D-21-00174)
Supplement: SUPPLEMENTARY MATERIAL [file jagrr-5-e21.00174-s002.pdf]

The CORR Procedure

**1 With Variables:**	NRS
**1 Variables:**	IPA

Simple Statistics

Variable	N	Mean	Std Dev	Median	Minimum	Maximum	Label
**NRS**	322	5.66460	3.24866	6.00000	0	10.00000	NRS
**IPA**	322	1.18944	0.65909	1.00000	0	2.00000	IPA

Spearman Correlation Coefficients, N = 322
Prob > |r| under H0: Rho=0

	**IPA**
**NRS**	0.65910
NRS	<.0001

Kendall Tau b Correlation Coefficients, N = 322
Prob > |tau| under H0: Tau=0

	**IPA**
**NRS**	0.57666

NRS	<.0001

Hoeffding Dependence Coefficients, N = 322
Prob > D under H0: D=0

	**IPA**
**NRS**	0.11152
NRS	<.0001

Association of IPA with time

The CORR Procedure

**1 With Variables:**	Time
**1 Variables:**	IPA

Simple Statistics

Variable	N	Mean	Std Dev	Median	Minimum	Maximum	Label
**Time**	192	30.79867	62.52713	12.00000	0	468.00000	Time
**IPA**	322	1.18944	0.65909	1.00000	0	2.00000	IPA

Kendall Tau b Correlation Coefficients
Prob > |tau| under H0: Tau=0
Number of Observations

	IPA
**Time**<br>Time	0.01181<br>**0.8403**<br>192

Association between NRS and time

The CORR Procedure

**1 With Variables:**	Time
**1 Variables:**	NRS

Simple Statistics

Variable	N	Mean	Std Dev	Median	Minimum	Maximum	Label
**Time**	192	30.79867	62.52713	12.00000	0	468.00000	Time
**NRS**	322	5.66460	3.24866	6.00000	0	10.00000	NRS

Kendall Tau b Correlation Coefficients
Prob > |tau| under H0: Tau=0
Number of Observations

	**NRS**
**Time**	0.03390
Time	**0.5215**
	192
